# On the Success of the Hadal Snailfishes

**DOI:** 10.1093/iob/obz004

**Published:** 2019-03-23

**Authors:** M E Gerringer

**Affiliations:** Friday Harbor Laboratories, University of Washington, Friday Harbor, WA 98250, USA

## Abstract

Deep-sea trenches, depths 6000–11,000 m, are characterized by high pressures, low temperatures, and absence of sunlight. These features make up the majority of the deepest marine habitat—the hadal zone—home to distinct communities from those in the surrounding abyssal plains. The snailfishes, family Liparidae (Scorpaeniformes), have found notable success in the hadal zone from ∼6000 to 8200 m, comprising the dominant ichthyofauna in at least six trenches worldwide. The hadal fish community is distinct from the abyssal community where elongate, scavenging fishes such as rattails (Macrouridae), cutthroat eels (Synaphobranchidae), tripodfishes (Ipnopidae), eelpouts (Zoarcidae), and cusk eels (Ophidiidae) are most common. Until recently, little was known about the biology of these deepest-living fishes, or the factors that drive their success at hadal depths. Here, I review recent investigations spanning the abyssal–hadal boundary and discuss the factors structuring these communities, including the roles of pressure adaptation, feeding ecology, and life history. Hadal fishes show specialized adaptation to hydrostatic pressure both in accumulation of the pressure-counteractant trimethylamine *n*-oxide and in intrinsic changes to enzymes. Stomach content and amino acid isotope analyses, and jaw morphology suggest that suction-feeding predatory fishes like hadal liparids may find an advantage to descending into the trench where amphipods are increasingly abundant. Analysis of otolith growth zones suggest that snailfishes may be adapted to a seismically active, high-disturbance hadal environment by having relatively short life-spans. This review synthesizes the known literature on the planet’s deepest-living fishes and informs new understanding of adaptations to life in the trenches.

## Synopsis

Sobre el éxito de los Peces Caracol Las fosas marinas con profundidades de 6000 a 11.000 m se caracterizan por altas presiones, bajas temperaturas, y ausencia de luz solar. Estas características constituyen la mayoría de los hábitats marinos más profundos, la zona hadal, que albergan distintas comunidades que rodean a las planices abisales. Los peces caracol de la familia Liparidae (Scorpaeniformes) conforman la ictiofauna dominante en zonas de 6000 a 8200 m y se encuentran en al menos 6 fosas a nivle mundial. La comunidad de peces caracol es distinta de la comunidad abisal en esta última los peces tienen cuerpos más elongados y suelen ser saprófitos como los peces cola de rata (Macrouridae), las anguilas de las familias Synaphobranchidae, Zoarcidae, Ophidiidae. Hasta hace poco, la imformación acerca de la biología de estos peces de profundidad era casi nula así como los factores que impulsan su éxito en las profundidades. El objetivo de este artículo es hacer una revisión de las investigaciones recientes que abarcan el límite abisal-hadal y discutir los factores que estructuran estas comunidades, incluidos los roles de adaptación a la presión, ecología de la alimentación e historia de la vida. Los peces caracil muestran una adaptación especializada a la presión hidrostática, tanto en la acumulación del *n*-óxido de trimetilamina del agente de presión como en los cambios intrínsecos a las enzimas. El contenido del estómago y los análisis de isótopos de aminoácidos, y la morfología de la mandíbula sugieren que los peces depredadores que se alimentan por succión, como los lipáridos, pueden encontrar una ventaja al descender a la zanja, donde los anfípodos son cada vez más abundantes. El análisis de las zonas de crecimiento de los otolitos sugiere que los peces caracol se pueden adaptar a un ambiente hadal sísmicamente activo y de alta perturbación al tener vidas relativamente cortas. Esta revisión sintetiza la literatura conocida sobre los peces de vida más profunda del planeta e informa una nueva comprensión de las adaptaciones a la vida en las trincheras. Translated to Spanish by S. Hinojosa (hinojosa.silvia@gmail.com)

Sobre o Sucesso dos Snailfishes Hadal As fossas oceânicas profundidas (do inglês “deep-sea trenches”), cujas profundidades variam entre 6000 e 11.000 m, são caracterizadas por altas pressões, baixas temperaturas, e ausência de luz solar. Esses padrões caracterizam a maior parcela dos habitat marinho mais profundos—a zona hadal—região que abriga comunidades distintas se comparadas às planícies abissais adjacentes. Os peixe-caracóis, família Liparidae (Scorpaeniformes), obtiveram notável sucesso na zona hadal entre 6000–8200 m, faixa que abriga a ictiofauna dominante em pelo menos seis fossas distintasa ao redor do mundo. A comunidade de peixes hadais é distinta da comunidade abissal, onde carniceiros de corpo elongado, como os peixes granadeiros (Macrouridae), Sinafobranquídeos, peixes-tripé (Ipnopidae), Zoarcídeos, e cobrelos (Ophidiidae) são os mais comuns. Até recentemente, pouco se sabia sobre a biologia desses peixes que habitam regiões mais profundas, ou os fatores que impulsionam seu sucesso na em profundidades hadais. Aqui, eu reviso a literatura recente que compreende a fronteira abissal–hadal e discuto os fatores que estruturam essas comunidades, incluindo os papéis da adaptação à pressão, ecologia de alimentação e história de vida. Os peixes da regiao hadal apresentam uma adaptações específicas à pressão hidrostática, tanto no acúmulo do neutraliante de pressão *N*-óxido de trimetilamina quanto em alterações intrínsecas em enzimas. As análises de conteúdo estomacal e isótopos de aminoácidos e a morfologia mandibular sugerem que peixes predadores com alimentação por sucção, como os liparídeos da zona hadal, podem encontrar uma vantagem ao descerem para as fossas, onde anfípodes são cada vez mais abundantes. A análise das zonas de crescimento dos otólitos sugerem que os peixes-caracóis podem estar adaptados a ambientes hadais altamente perturbados e sismicamente ativos por terem longevidade relativamente curta. Esta revisão sintetiza a literatura conhecida sobre os peixes dos ambientes mais profundos do planeta e apresenta uma nova interpretação das adaptações à vida em fossas oceânicas. Translated to Portuguese by J.P. Fontenelle (jp.fontenelle@mail.utoronto.ca)

Über den Erfolg der Scheibenbäuche des Hadals Tiefseegräben mit einer Tiefe von 6000–11.000 m zeichnen sich durch hohe Drücke, niedrige Temperaturen, und der Abwesenheit von Sonnenlicht aus. Diese Eigenschaften machen den Großteil des tiefsten Lebensraums der Meere—der Hadal-Zone—aus, wo Gemeinschaften zu Hause sind, die sich distinkt von jenen in den umliegenden Abyssal–Ebenen unterscheiden. Die Scheibenbäuche, Familie Liparidae (Scorpaeniformes), haben in der Hadal-Zone von ∼6000–8200 m einen beachtlichen Erfolg erzielt, sie stellen die dominierende Ichthyofauna in mindestens sechs Gräben weltweit. Die Gemeinschaft der Fische des Hadals unterscheidet sich von der abyssischen Gemeinschaft, in der vor allem längliche, aasfressende Fische wie Grenadierfische (Macrouridae), Grubenaale (Synaphobranchidae), Netzaugenfische (Ipnopidae), Aalmuttern (Zoarcidae) und Bartmännchen (Ophidiidae) häufig sind. Bis vor kurzem war wenig über die Biologie dieser am tiefsten lebenden Fische bekannt oder über die Faktoren, die ihren Erfolg in den Tiefe des Hadals beeinflussen. Ich bespreche hier jüngste Untersuchungen, unter Einbezug der Grenze zwischen dem Abyssal und dem Hadal, und diskutiere dabei die Faktoren welche diese Gemeinschaften strukturieren, einschließlich der Rolle von Druckanpassungen, Nahrungsökologie und Life History. Fische des Hadals zeigen eine spezialisierte Anpassung an den hydrostatischen Druck sowohl durch die Ansammlung des druckgegenwirkenden Trimethylamin-*n*-oxids als auch durch intrinsische Veränderungen von Enzymen. Analysen von Mageninhalt und Aminosäure-Isotopen sowie die Morphologie des Kiefers legen nahe, dass Raubfische, welche ihre Nahrung durch Ansaugen aufnehmen, wie Hadal-Lipariden, einen Vorteil durch den Abstieg in den Graben haben könnten, wo Amphipoden zunehmend häufiger sind. Die Analyse der Otolith-Wachstumszonen lässt vermuten, dass Scheibenbäuche an eine seismisch aktive, viel gestörte Umwelt im Hadal durch eine relativ kurze Lebensdauer angepasst sein könnten. Dieser Aufsatz fasst die bekannte Literatur über die am tiefsten lebenden Fische des Planeten zusammen und vermittelt ein neues Verständnis von Anpassungen an das Leben in den Tiefseegräben. Translated to German by F. Klimm (frederike.klimm@neptun.uni-freiburg.de)

## Introduction

**Fig. 1:**
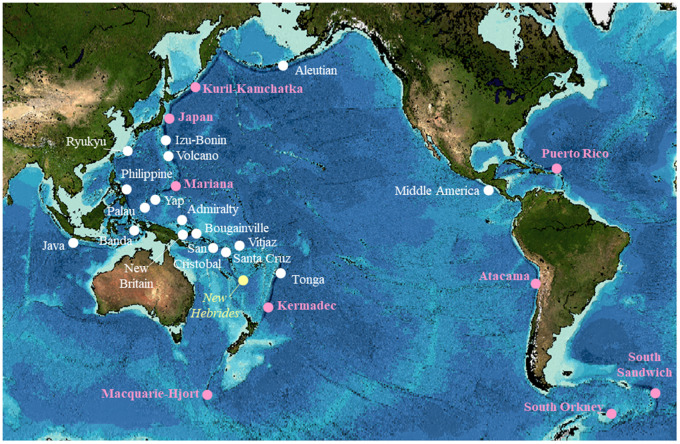
Hadal subduction trenches with depths exceeding 6000 m. Constructed using bathymetry data from the Global Multi-Resolution Topography (GMRT) synthesis data set (Ryan et al. 2009a) in GeoMapApp (www.geomapapp.org). List of trenches from Stewart and Jamieson (2018). Excludes 13 hadal trough and 7 trench fault habitats deeper than 6000 m (see map by Stewart and Jamieson [2018] for full hadal zone locations). Trenches with reports of snailfishes are highlighted in pink, trenches that have been sufficiently surveyed and shown to have no snailfishes shown in yellow.

The hadal zone—predominately deep-sea trenches with depths ranging from 6000 to 11,000 m—represents 45% of the ocean’s depth range but remains one of Earth’s least explored habitats (e.g., Jamieson 2015). Taking their name from the Greek underworld, these parts of the ocean are characterized by conditions of high hydrostatic pressures, low temperatures, and complete absence of sunlight (Bruun 1957). The hadal zone is made up of 47 distinct habitats, including 27 subducting trenches, most of which are located around the Pacific Rim (Fig. 1; Stewart and Jamieson 2018). Trenches are long, narrow features, reaching lengths up to 4500 km and usually less than 100 km wide, made up of complex habitats including steep slopes and sedimentary basins (Stewart and Jamieson 2018).

Many of the factors controlling the physiology and ecology of organisms found in hadal trenches are similar to those in the surrounding abyss and broader deep sea. However, a few exceptions make the hadal zone a distinct environment, most notably increased hydrostatic pressure, high levels of seismicity at plate boundaries, and a sloping topography distinct from the surrounding abyssal plains. With these environmental conditions, there are marked faunal transitions from the abyss to the hadal zone (Wolff 1970; Jamieson 2011). The hadal faunal community includes amphipods, tanaids, isopods, cumaceans, decapods, echinoderms, nematodes, polychaetes, copepods, molluscs, foraminifera, cnidarians, and fishes, at apparently high levels of endemism (Wolff 1958; Beliaev 1989; Jamieson et al. 2010; Jamieson 2015).

**Fig. 2:**
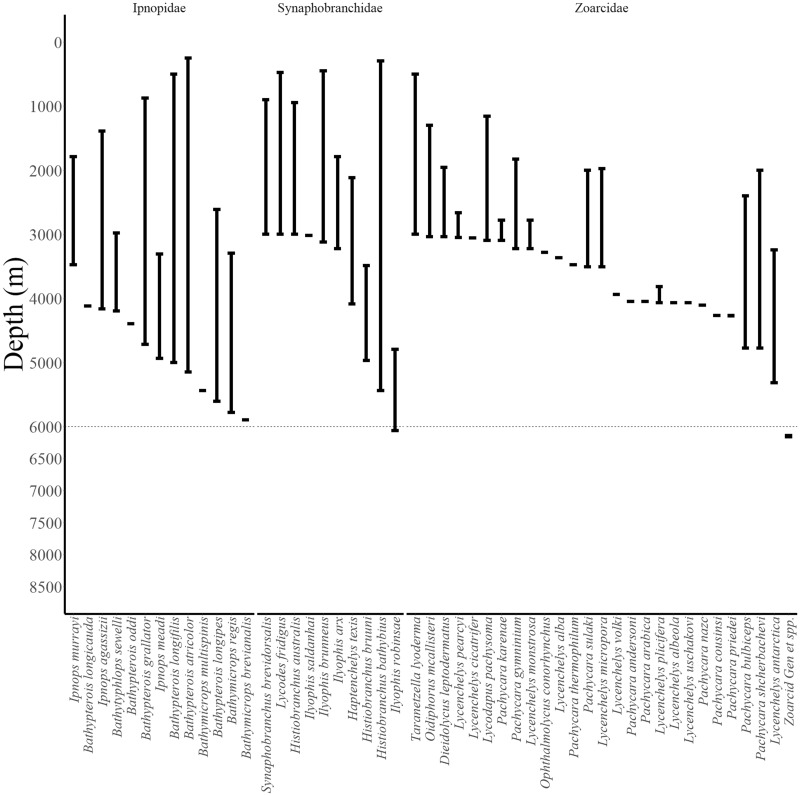
Reported depth distributions of deep-dwelling fishes (collections >3000 m) by family (Ipnopidae, Synaphobranchidae, Zoarcidae). Abyssal–hadal boundary at 6000 m shown as dotted line. Data from FishBase (Froese and Pauly 2014) checked in the primary literature (Nielsen 1977; Andriashev and Stein 1998; Nielsen et al. 1999; Chernova et al. 2004; Chernova 2006; Duhamel and King 2007; Moller and King 2007; Jamieson et al. 2009; Linley et al. 2016, 2017; Gerringer et al. 2017b). See Supplementary Note 1 for notes on record verifications.

**Fig. 3:**
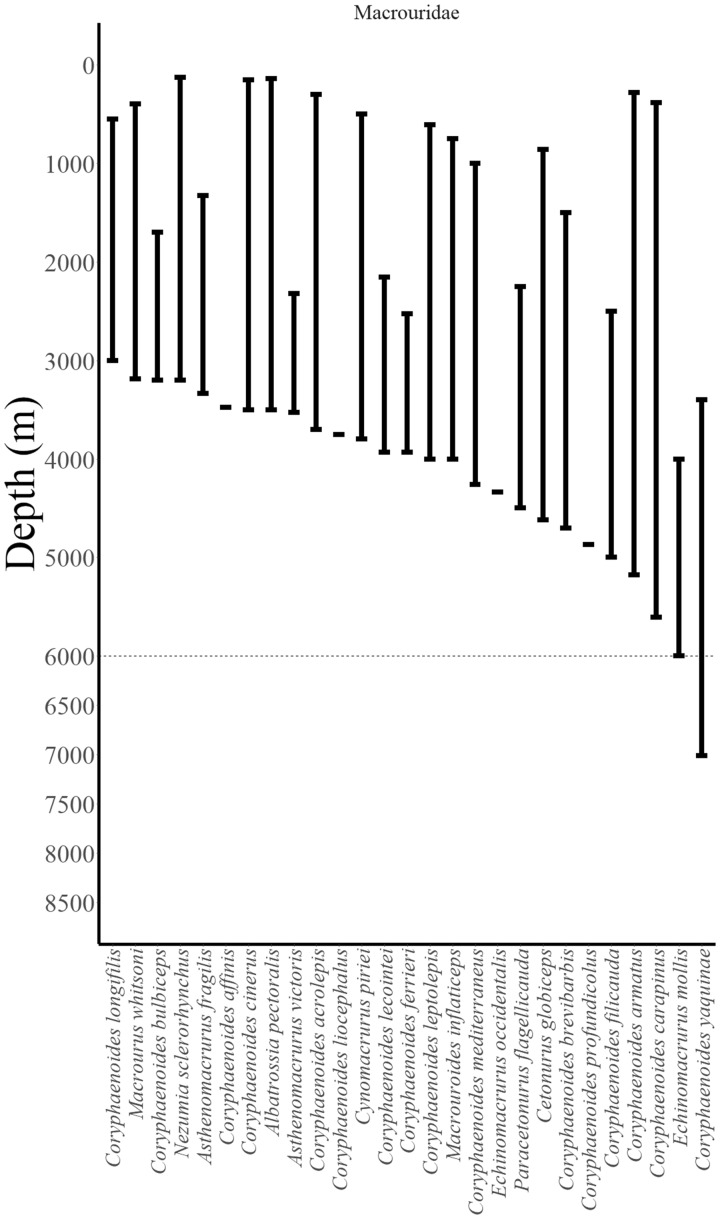
Reported depth distributions of deep-dwelling fishes (collections >3000 m) by family (Macrouridae). Abyssal–hadal boundary at 6000 m shown as dotted line. Data from FishBase (Froese and Pauly 2014) checked in the primary literature (Nielsen 1977; Andriashev and Stein 1998; Nielsen et al. 1999; Chernova et al. 2004; Chernova 2006; Duhamel and King 2007; Moller and King 2007; Jamieson et al. 2009; Linley et al. 2016, 2017; Gerringer et al. 2017b). See Supplementary Note 1 for notes on record verifications.

**Fig. 4:**
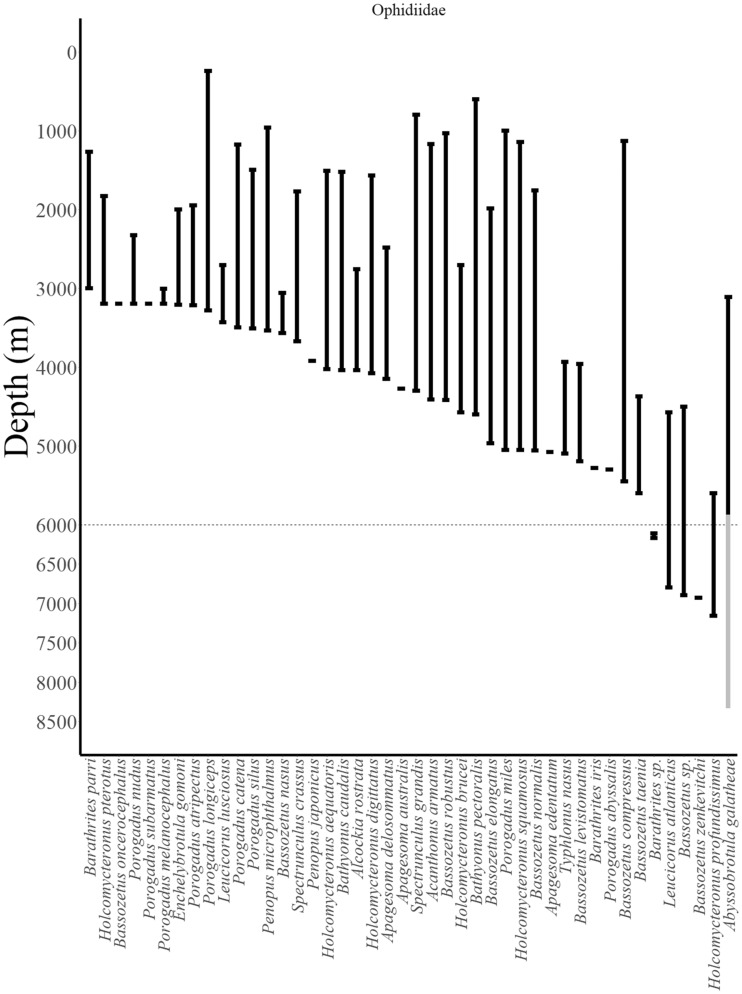
Reported depth distributions of deep-dwelling fishes (collections >3000 m) by family (Ophidiidae). Abyssal–hadal boundary at 6000 m shown as dotted line. Data from FishBase (Froese and Pauly 2014) checked in the primary literature (Nielsen 1977; Andriashev and Stein 1998; Nielsen et al. 1999; Chernova et al. 2004; Chernova 2006; Duhamel and King 2007; Moller and King 2007; Jamieson et al. 2009; Linley et al. 2016, 2017; Gerringer et al. 2017b). Uncertain record from open trawl shown in gray. See Supplementary Note 1 for notes on record verifications.

**Fig. 5:**
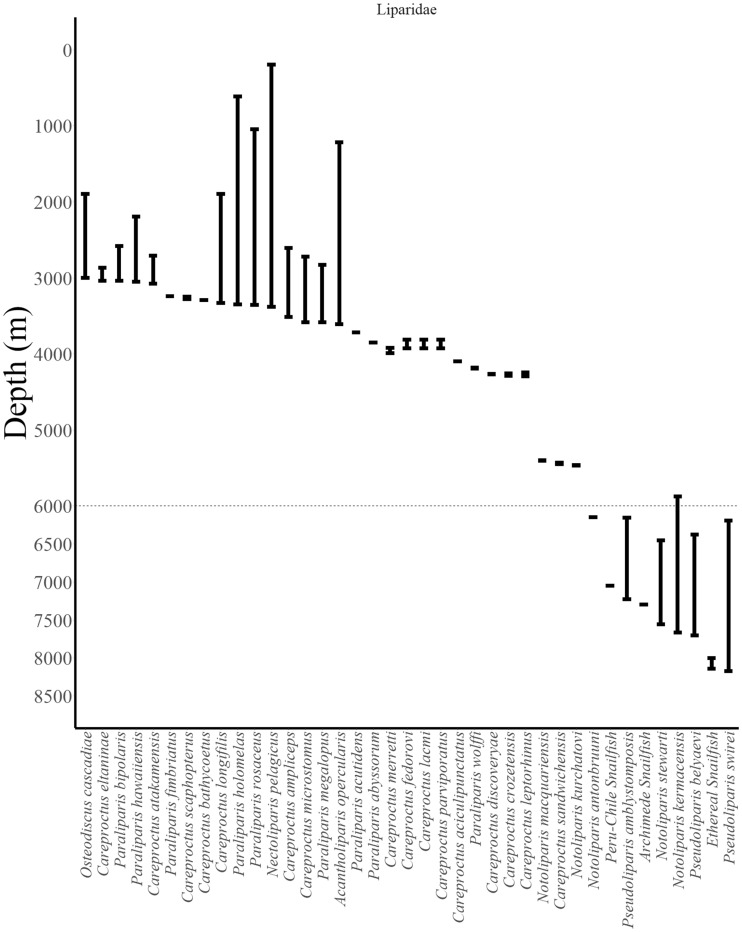
Reported depth distributions of deep-dwelling fishes (collections >3000 m) by family (Liparidae). Abyssal–hadal boundary at 6000 m shown as dotted line. Data from FishBase (Froese and Pauly 2014) checked in the primary literature (Nielsen 1977; Andriashev and Stein 1998; Nielsen et al. 1999; Chernova et al. 2004; Chernova 2006; Duhamel and King 2007; Moller and King 2007; Jamieson et al. 2009; Linley et al. 2016, 2017; Gerringer et al. 2017b). Excludes the liparids *Psednos griseus* and *P. melanocephalus*, which are each known only from one individual, collected somewhere between 0 and 4000 m depth (Chernova and Stein 2002).

**Fig. 6:**
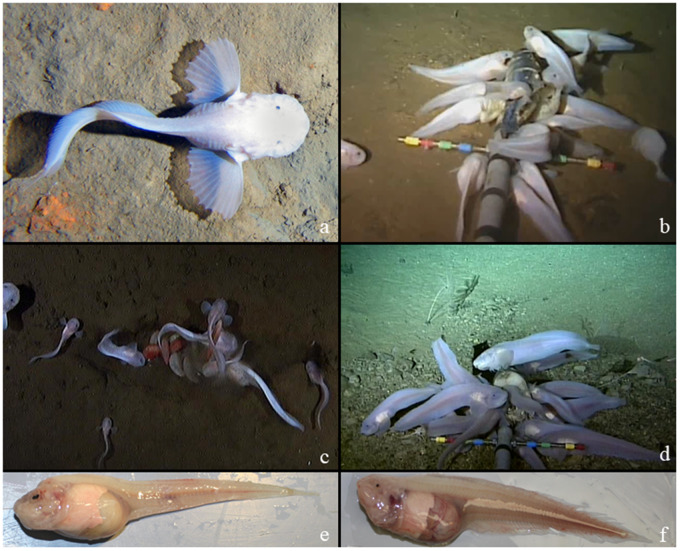
Hadal liparids. *In situ* photographs of hadal snailfishes. **a**) c.f. *Notoliparis antonbruuni*, Atacama Trench, **b**) *Pseudoliparis swirei*, Mariana Trench, **c**) *Pseudoliparis belyaevi*, Japan Trench, **d**) *Notoliparis kermadecensis*, Kermadec Trench. Photos by Alan Jamieson, Newcastle University. Collections of hadal snailfish from the Mariana (**e**) and Kermadec (**f**) trenches, Hadal Ecosystem Studies cruises.

The first fish discovered from hadal depths was the cusk eel, *Bassogigas profundismus* (family Ophidiidae), caught in 1901 from 6035 m in the Moseley Trench by the Princess Alice expedition (Nielsen 1964). With additional exploration, however, a distinct shift in the fish community at the abyssal–hadal boundary was discovered (Figs. 2–5; Supplementary Note 1). Snailfishes (Liparidae, Scorpaeniformes, Fig. 6) now appear to be the most common vertebrates in the hadal environment. The shift from the abyssal to hadal fish community is an overlapping transition over about 500 m depth (Jamieson et al. 2011). For example, the macrourid *Coryphaenoides yaquinae* can, on occasion, be seen as deep as 7000 m, but they are most commonly found at depths less than 6000 m (Endo and Okamura 1992; Linley et al. 2017).) In 1952, on the Danish *Galathea II* expedition—one of two prominent early voyages of hadal research—five individuals of the hadal snailfish *Notoliparis kermadecensis* (Nielsen 1964) were caught between 6660 and 6770 m in the Kermadec Trench (Nielsen 1964). The other pioneering hadal expeditions, conducted aboard the Soviet vessel *Vityaz*, collected the snailfish, *Pseudoliparis amblystomopsis* (Andriashev 1955) in the Kuril–Kamchatka Trench at 7230 m in 1955 and in the Japan Trench to a maximum depth of 7579 m in 1957 (Nielsen 1964). Other hadal and deep-abyssal snailfishes were seen in the South Sandwich Trench, *Careproctus sandwichensi*s at 5435–5453 m (Andriashev and Stein 1998), and in the Peru–Chile Trench (Fujii et al. 2010). Historical collections of hadal liparids have been few and far between (Table 1), limiting our understanding of the biology and ecology of these fishes.

**Table 1:** Global collections of hadal liparids by year

Trench	Species	Year	Depth	Ship	*n*
Kurile-Kamchatka	*Pseudoliparis amblystomopsis*	1953	7230	*Vitjaz*	1
Japan	*Pseudoliparis amblystomopsis*	1955	6156–7579	*Vitjaz*	5
Japan	*Pseudoliparis belyaevi*	1957	7579	*Vitjaz*	1
Kermadec	*Notoliparis kermadecensis*	1952	6660–6770	*Galathea*	5
Peru-Chile	*Notoliparis antonbruuni*	1966	6150	*Anton Bruun*	1
Japan	*Pseudoliparis amblystomopsis*	2009	7000	*Hakuho maru*	2
Kermadec	*Notoliparis kermadecensis*	2011	7002–7050	*Kaharoa*	6
Kermadec	*Notoliparis stewarti*	2011	7000	*Kaharoa*	1
Kermadec	*Notoliparis kermadecensis*	2014	6456–7554	*Thompson*	35
Kermadec	*Notoliparis stewarti*	2014	6456–7560	*Thompson*	6
Mariana	*Pseudoliparis swirei*	2014	6898–7966	*Falkor*	37
Mariana	*Pseudoliparis swirei*	2017	7581	*Shinyo-maru*	1

Collection year noted, depth presented in meters, *n* indicates the number of specimens collected. Current as of January 1, 2018. Ships operated out of the former Soviet Union, Denmark, USA, Japan, and New Zealand. Total: 101 specimens.

As a family, snailfishes are found in a wide variety of habitats, from the intertidal to the hadal environment and from polar systems (Matallanas and Pequeno 2000) to the subtropics (Chernova et al. 2004). They are typically small, tadpole-shaped fishes which live in temperate to cold waters. Snailfishes display an array of interesting adaptations to distinct marine environments, including an antifreeze protein in the skin of Antarctic species (Evans and Fletcher 2001; Hobbs and Fletcher 2013), ossicles that may offer protection with minimum additional weight (Märss et al. 2010), and a subdermal extracellular matrix for increasing buoyancy and maintaining ideal body shape for drag reduction (Eastman et al. 1994; Gerringer et al. 2017a). The snailfishes are an understudied group, with many new species still being described (Chernova et al. 2004; Orr 2004; Chernova and Møller 2008; Stein and Drazen 2014) and with several species-level identifications and generic allocations still contentious (Kai et al. 2011; Orr et al., Accepted Manuscript.).

The abundance and significance of the family Liparidae at hadal depths (Table 2) has become increasingly clear through recent discoveries (e.g., Linley et al. 2016; Gerringer et al. 2017b). There are currently 12 species of hadal and near-hadal liparids (nine formally described, three observed) known from nine different trenches (Table 2). This marks a noticeable shift in the fish community at the abyssal–hadal boundary: from elongate, cosmopolitan species of the families Macrouridae (rattails), Ophidiidae (cusk eels), Ipnopidae (tripodfishes), Synaphobranchidae (cutthroat eels), and Zoarcidae (eelpouts) on the abyssal plains (Wilson and Waples 1983; Nielsen and Merrett 2000; Milligan et al. 2016), to the hadal snailfishes (Liparidae) in the trenches. This review synthesizes the current scientific literature to address the following question: What evolutionary drivers have factored into the success of snailfishes in the trench environment relative to other potential colonizers?

**Table 2:** Global geographic and bathymetric distribution of hadal liparids from observations and collections

Trench	Depth (m)	Species	Reference
Japan Trench	7420–7450	*Pseudoliparis amblystomopsis*	(Andriashev 1955)
	6380–7703	*Pseudoliparis belyaevi*	(Andriashev and Pitruk 1993; Fujii et al. 2010)
Kermadec Trench	5879–7669	*Notoliparis kermadecensis*	(Linley et al. 2016)
	6456–7560	*Notoliparis stewarti*	(Stein 2016)
Kuril-Kamchatka	6156–7587	*Pseudoliparis amblystomopsis*	(Andriashev 1955)
Macquarie-Hjort Trench	5400–5410	*Notoliparis macquariensis*	(Andriashev 1978)
Mariana Trench	6198–8178	*Pseudoliparis swirei*	(Gerringer et al. 2017b; Oguri and Noguchi 2017)
	8007–8145	Ethereal snailfish	(Linley et al. 2016)
Peru-Chile Trench	6150	*Notoliparis antonbruuni*	(Stein 2005)
	7049	Peru–Chile snailfish	(Linley et al. 2016)
Puerto Rico Trench	7300	Archimede snailfish	(Pérês 1965)
South Orkney Trench	5465–5474	*Notoliparis kurchatovi*	(Andriashev 1975)
South Sandwich Trench	5435–5453	*Careproctus sandwichensis*	(Andriashev and Stein 1998)

Includes records of abyssal species reported near trenches, current as of January 1, 2018. HOV *Archimede* observation in Puerto Rico Trench is probable, but anecdotal, see text for details. Undescribed species listed with common names.

## Adaptations to high hydrostatic pressure

Perhaps the most intuitive factor that influences bathymetric ranges is hydrostatic pressure, long understood to limit radiation into the deep sea (Günther 1887). In the marine environment, pressures increase 1 atm with every 10 m depth, reaching as high as 1100 times atmospheric pressure (110 megapascals, MPa) at the ocean’s deepest point. Naturally, only organisms that have adapted to high hydrostatic pressures are able to inhabit the hadal zone. Adaptations to high pressure occur on multiple scales, from the organismal to the molecular, from the limiting of gas-filled spaces such as swim bladders (Scholander 1954; Priede 2018) to homeoviscous adaptations to maintain cellular membrane fluidity under pressure through an increase in polyunsaturated fatty acid compositions (Sinenesky 1974; Somero 1992).

Hydrostatic pressure is believed to set a depth limit for marine fishes nearly 2000 m shy of full-ocean depth. The depth limit hypothesis proposed by Yancey et al. (2014) centers on the pressure-counteracting osmolyte trimethyl-amine oxide (TMAO). TMAO, a byproduct of lipid metabolism, increases concentration linearly with increasing habitat depth in fishes (Kelly and Yancey 1999). It appears that fishes living at greater pressures require TMAO to stabilize their proteins, a process thought to occur through the alteration of solvent interactions (e.g., Yancey and Siebenaller 1999). As the TMAO concentration increases, cellular osmolality also increases. At a certain depth—around 8200 m (Yancey et al. 2014)—fish cells contain such high TMAO concentrations that they reach the same osmolality as seawater. Adding additional TMAO to go deeper would pose an osmoregulatory problem, requiring a physiological shift to hyperosmotic life (Yancey et al. 2014). Hadal liparids have high TMAO concentrations and cellular osmolalities near isosmotic with seawater, suggesting that they are living at near the maximum depth for marine fishes (Yancey et al. 2014; Linley et al. 2016).

The accumulation of pressure-stabilizing molecules (piezolytes) such as TMAO represents an extrinsic adaptation, which alters the cellular milieu for protein function (reviewed by Yancey and Siebenaller 2015). However, organisms living under high hydrostatic pressures also exhibit intrinsic adaptations, where specific protein structures differ from those of their shallow-living counterparts. Several well-known pressure adaptations in other deep-sea taxa—such as high concentrations of polyunsaturated fatty acids to maintain membrane fluidity (Cossins and Macdonald 1986), reduced pressure sensitivities in Na^+^–K^+^–ATPases (Gibbs and Somero 1989), and adaptations of muscle actins (Swezey and Somero 1982)—likely allow hadal liparids to survive under pressure, although these adaptations have not yet been investigated to hadal depths. Gerringer et al. (2017c) detailed a newly-discovered pressure adaptation in abyssal and hadal species—intrinsic changes in lactate dehydrogenases of hadal liparids and abyssal macrourids that allow the enzymes to function better (increased maximum reaction rate, *V*_max_) at *in situ* pressures than they do at atmospheric pressure. Maximum reaction rates of lactate dehydrogenases, which catalyze the conversion of pyruvate to lactate in anaerobic glycolysis, in hadal liparids increased under habitat pressures of 60 MPa (water depth 6000 m). In shallow-living fishes, this enzyme was pressure inhibited. Adaptations to high pressure also occur at the organismal level in hadal liparids, which exhibit high volumes of gelatinous tissues and watery muscles to maintain buoyancy under high pressures without a gas bladder (Gerringer et al. 2017a). The scope and diversity of pressure adaptations is great, and many more significant findings await in the study of hadal taxa.

## Feeding ecology

The v-shaped topography of hadal trenches is thought to funnel organic matter into the hadal zone, resulting in higher food availability for hadal organisms than for those in the food-limited abyss (George and Higgins 1979; Danovaro et al. 2003; Jamieson 2011; Ichino et al. 2015). This funneling is enhanced because hadal trenches are usually subduction zones—sites of high seismic activity that can trigger turbidity flows—bringing substantial quantities of sediment and organic matter into the trench (Itou et al. 2000; Oguri et al. 2013). Increased organic matter concentrations in the hadal zone seem to be accompanied by higher faunal abundances and oxygen consumption that at abyssal depths (Beliaev 1989; Danovaro et al. 2002; Itoh et al. 2011; Glud et al. 2013; Wenzhöfer et al. 2016).

In hadal trenches, amphipod abundance increases substantially with increasing depth (Jamieson 2015), an effect that likely impacts the fish community (Gerringer et al. 2017d). Snailfishes may have a nutritional advantage in hadal trenches due to increased abundances of small crustacean prey such as amphipods (Gerringer et al. 2017d). Fishes that rely on piscivory or scavenging, such as macrourids or synaphobranchids (reviewed by Drazen and Sutton 2017), might not have the same evolutionary pressures to invade hadal depths. Carrion is just as likely to fall in a trench as anywhere on the abyssal plains, indeed, far less likely considering the small global area of trenches compared with abyssal depths. Stomach contents and δ^15^N values of specific amino acids for two hadal snailfishes (*N. kermadecensis* and *P. swirei*) showed that amphipods are the most important prey item in both species (Gerringer et al. 2017d). This study revealed additional predator–prey relationships in the hadal zone—particularly that hadal liparids eat decapods, and, in some species, swimming polychaetes. Stomach content compositions and trophic positions were distinctly different for the abyssal and hadal species examined due differences in both feeding strategies and food availability.

**Fig. 7:**
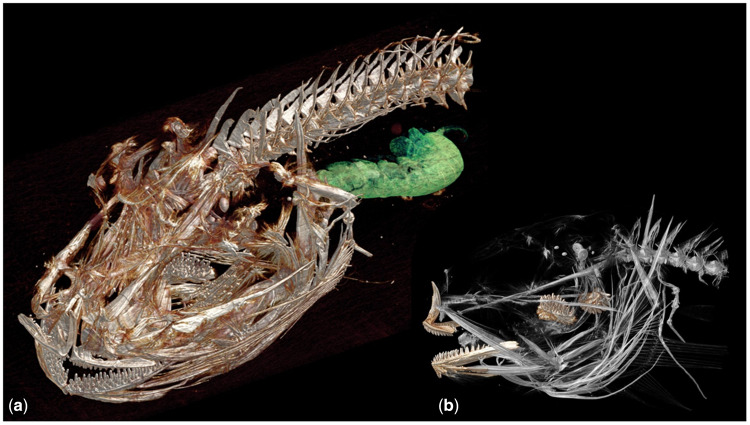
**a)** Skeletal morphology of holotype hadal snailfish, *P. swirei*, from micro-computed tomography. Amphipod prey visible in the stomach in green. Fish head length 18.9 mm. **b**) The strong pharyngeal jaw apparatus plays an important role in amphipod feeding.

A detailed functional morphology of abyssal and hadal fish feeding, similar to what has been conducted for Antarctic fishes (Bansode et al. 2014), may also further illuminate the role of trophic ecology in niche partitioning and depth zonation in the deep ocean. In addition to a shift in feeding strategy—from certain abyssal fishes that bite and tear their food to suction-feeding in hadal fishes—functional morphology and modeling of suction feeding parameters may reveal differences in optimum prey sizes for abyssal and hadal species. A difference in optimum prey size in relation to prey availability may partially account for the abundance of liparids in the hadal zone in comparison to the ophidiids, which also suction feed but have only a shallow-hadal and abyssal range (Linley et al. 2017). Further, the strong pharyngeal jaw apparatus (Fig. 7) found in snailfishes from the Kermadec and Mariana trenches (Gerringer et al. 2017d) likely contributes to their ability to thrive on the large amphipod biomass at hadal depths.

**Fig. 8:**
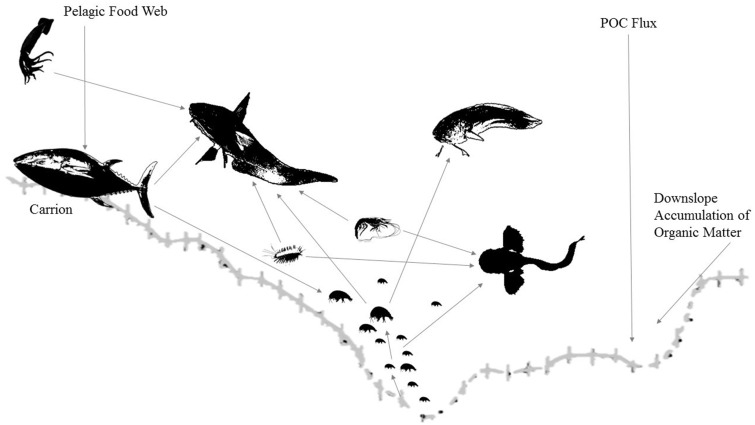
Generalized hadal food web derived from information presented here. Bathymetry based on Japan Trench (Fisher 1954). Arrows indicate known trophic linkages. Organisms shown include squid, macrourids, ophidiids, liparids, decapods, polychaetes, and amphipods. Macrourids and ophidiids are part of the abyssal/upper edges of the hadal food web. POC, particulate organic carbon. Drawings based on photos from MBARI, NOAA OER, Stuart Piertney, Alan Jamieson. Not to scale.

Although few data exist on feeding ecology at hadal depths (Blankenship and Levin 2007; Gerringer et al. 2017d), recent studies have informed new understanding of trophic linkages in the hadal zone. With additional analyses of stomach contents, stable isotopic compositions, and *in situ* feeding observations, the goal of assembling a hadal food web will be within reach. Further research is necessary to construct a complete food web; however, a preliminary outline is included in Fig. 8. Additional stomach contents and stable isotope analysis on other taxa—particularly amphipods, decapods, and holothurians—would be needed. Understanding the hadal food web is important not only as a matter of ecological interest, but also for tracking global patterns of carbon turnover. The hypothesis that trenches act as sinks of organic matter has been largely accepted (George and Higgins 1979; Danovaro et al. 2002; Itoh et al. 2011; Glud et al. 2013; Ichino et al. 2015). Many authors note that accumulation of organic material would be significant for global carbon cycling, but the details of this process require further research (Oguri et al. 2013; Wenzhöfer et al. 2016).

## Life history

**Fig. 9:**
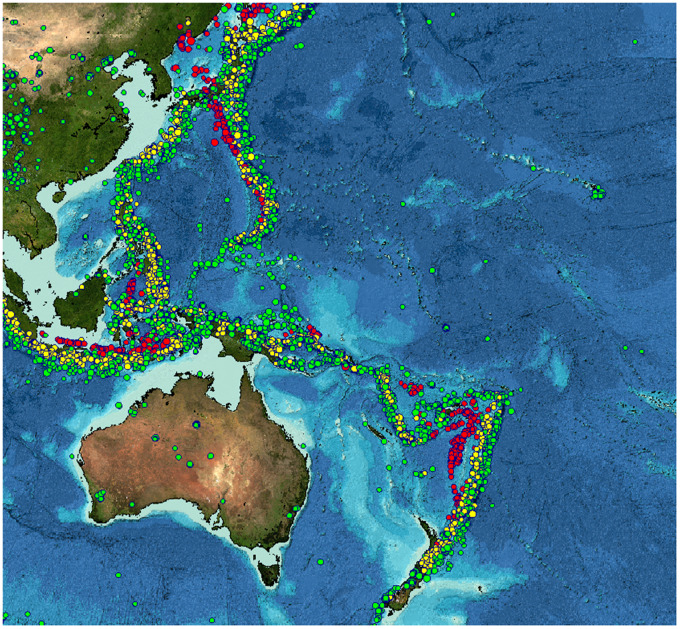
Foci of large earthquakes (magnitude 5.0–9.0) in the Western Pacific over a 30-year period (1964–1995), showing hadal zones to be areas of high seismic activity. Earthquake data from the International Seismological Centre. Bathymetry from General Bathymetric Chart of the Oceans (GEBCO) and GMRT (Ryan et al. 2009b), map made in GeoMapApp (www.geomapapp.org). Points are colored by earthquake depth: <50 km (green), 50–250 km (yellow), and >250 km (red).

Hadal trenches are high-disturbance environments. Most trenches are located on subduction zones, sites of high seismic activity (Fig. 9). These seismic events can trigger rapid downslope movements of sediment known as turbidity flows, akin to undersea avalanches (e.g., Stewart and Jamieson 2018). The dramatic effect that turbidity flows can have on the hadal community was witnessed firsthand by *in situ* observations in the Japan Trench shortly after the Tohoku-Oki earthquake. Large nepheloid layers tens of meters above the seafloor were present, even four months after the earthquake. Dead organisms were seen in high numbers, with sediment cores showing evidence of additional recent mass-wasting events (Oguri et al. 2013). We often consider the deep sea to be a stable environment, but hadal trenches, like hydrothermal vents, mark noteworthy exceptions.

Deep-sea fishes are often considered to be long-lived (Cailliet et al. 2001; Drazen and Haedrich 2012), which might not be suited to high-disturbance environments like hadal trenches. Based on opaque growth zones in sagittal otoliths, hadal liparids do appear to be shorter-lived than other deep-sea species, with age estimates ranging from five to 16 years (Gerringer et al. 2018). These estimates are younger than those estimated for the shallower-living liparid *Careproctus melanurus*, which revealed up to 25 annuli (Gerringer et al. 2018), and are considerably lower than many deep-dwelling species (Cailliet et al. 2001; Drazen and Haedrich 2012). Hadal liparids also seem to exhibit continuous spawning, or at least periodic spawning on the sub-annual scale (Gerringer et al. 2017b, 2018). Both shorter lifespans and continuous spawning may be advantageous to a high-disturbance environment prone to seismic activity and subsequent destructive turbidity flows, allowing for their continued success in the hadal zone.

Future research should seek to validate these estimates and the age reading protocol. Although many methods for validation exist (reviewed by Campana 2001), most are not practical at either abyssal or hadal depths. Lead-radium dating may be used to validate the ages of abyssal rattails, *Coryphaenoides armatus* and *C. yaquinae*, as has been applied to other species in the Macrourid family (Andrews et al. 1999). However, this method requires nearly a gram of early otolith growth (core extraction of first few years) material for instrumental analysis. Due to the small size of the hadal liparid otoliths, multiple individuals from discrete size or age classes would be needed to pool enough material for lead-radium dating. This type of analysis would require the combination of multiple collections to cover a thorough size range, but would be a valuable way to verify growth patterns in a common and important abyssal group. Validated life history data on these deep-living species would also greatly inform discussions of the factors governing growth rate of deep-sea fishes (Cailliet et al. 2001; Drazen and Haedrich 2012), potentially clarifying the role of environmental variables such as temperature, pressure, and food supply. Other validation methods such as mark/recapture or outer ring analysis across multiple seasons are also impractical at this time (Gerringer et al. 2018).

It might be expected that hadal liparids would have benthic larvae—limiting dispersal and facilitating endemism. However, this was not supported by thermal history reconstructions based on oxygen isotopic composition of otoliths (Gerringer et al. 2018). Oxygen isotope ratios in otoliths vary with temperature and seawater δ^18^O, allowing thermal histories of fishes to be calculated from measurements across otolith growth zones (Thorrold et al. 1997). Unexpectedly, large changes in oxygen isotopic compositions suggest temperature changes of greater than 5°C across ontogeny for hadal liparids from both the Mariana and Kermadec trenches (Gerringer et al. 2018). If temperature is the only factor influencing the oxygen composition of hadal liparid otoliths, these results would reflect an approximately 5000 m depth differential between larval and adult populations, according to the established conversions (Thorrold et al. 1997; Høie et al. 2004; Chang et al. 2015). According to the published literature and validation efforts, this change could not be explained by metabolically-mediated fractionation, pressure effects, excess protein accumulation, or instrumental drift. It is possible that an alternative unknown effect on carbonate chemistry is taking place.

If these snailfishes are indeed feeding in shallow waters before returning to the hadal zone, this is an incredible feat. Large vertical migrations from larval habitat to adult habitat have been documented in other species, for example in macrourids up to 1400 m (Lin et al. 2012) and up to 800 m in the jellynose fish *Ateleopus japonicus* (Shiao et al. 2017). Further, snailfishes in the closely-related genus *Liparis* often exhibit planktonic larval stages (Marliave and Peden 1989; Sokolovskii and Sokolovskaya 2003; Walkusz et al. 2016) and thermal history reconstructions of the blacktail snailfish *C.melanurus* suggest similar life history traits (Gerringer et al. 2018). Although counterintuitive, a pelagic larval stage may also be supported by the interpretation of Munk (1964), who wrote “It seems fairly probable that all major constituents of the eyes of the deep-sea fishes examined have been fully differentiated before degeneration sets in, and it is suggested that the eyes may have been functionally normal in larvae and maybe also in young adults.” However, this would be one of the largest changes in hydrostatic pressure experienced by any metazoan and this finding certainly warrants further verification. Pressure effects, such as those explored in Gerringer et al. (2017c), would need to be investigated with this added complication of ontogenetic changes in habitat pressure. If hadal liparid larvae are indeed present at depths shallower than 1000 m (Gerringer et al. 2018), it should be possible to collect them by trawl, although similar surveys of deep-water snailfishes in Alaskan waters yielded few collections, despite the high number of known snailfish species in the area (Matarese et al. 2003). Although liparid larvae have few distinguishing characters (Kim et al. 1986a, 1986b; Marliave and Peden 1989) and confirming identifications morphologically would be difficult, genetic analysis could be used. This technique has been successfully applied in a number of systems for larval identification (e.g., Hubert et al. 2010; Riemann et al. 2010). Data from the genetic markers (mitochondrial genes 16S, COI, and Cyt-b) analyzed in Gerringer et al. (2017b) for *Notolipariskermadecensis* and *N. stewarti* from the Kermadec Trench and *Pseudoliparis swirei* from the Mariana Trench would allow for positive identification of hadal liparid larvae collected from shallower depths over the trench.

## Evolution into the hadal environment

The current literature shows hadal liparids to be highly-specialized, endemic fishes that thrive in deep-sea trenches through a suite of adaptations. Hadal liparids have been captured and described from the Kuril–Kamchatka Trench (Andriashev 1955), Japan Trench (Andriashev 1955; Andriashev and Pitruk 1993), Kermadec Trench (Nielsen 1964), and Peru–Chile Trench (Stein 2005). In addition to these known species, there are likely undiscovered species of hadal liparids in unexplored trenches. In 1964, for example, those diving the Puerto Rico Trench in the bathyscaphe *Archimede* reported that “at 7,300 m the community was characterized by the abundance of a liparidid fish (*Careproctus*?: about 200 individuals)” (Pérês 1965). Due to its geographic isolation from other hadal ecosystems, this finding would be of particular importance to the understanding of the adaptation and evolution of the planet’s deepest-living vertebrates. Megafauna collections in the Puerto Rico Trench would be a valuable target for future efforts, as would under-surveyed trenches such as the Aleutian, Philippine, and Java trenches.

The colonization of the deep sea by fishes, and by snailfishes in particular, is understood to be a radiation from shallow into the deep (Burke 1930; Priede and Froese 2013). Priede and Froese (2013) posit that the evolution of liparids into the trenches are the result of independent speciation events. However, recent genetic analyses (mtCOI) show that hadal liparid species included in recent phylogenetic analyses (*N. stewarti*, *N. kermadecensis*, *P. swirei*, and *P. belyaevi*) form a strongly supported clade together when compared with extant species (Gerringer et al. 2017b; Orr et al., Accepted Manuscript.). Further, Orr et al. (Accepted Manuscript) show that this clade is basal to nearly all other liparids. Future phylogenetic analyses of multiple hadal liparids, building on the trees presented by Gerringer et al. (2017b) and Orr et al. (Accepted Manuscript), would be valuable in understanding connectivity between hadal fish populations and evolutionary patterns of dispersal. One interesting hypothesis for future exploration is a potential Antarctic origin of hadal fishes. Researchers have long noticed similarities between the Antarctic, Arctic, and deep-sea habitats (e.g., Thorson 1950). These common evolutionary drivers including cold temperatures and prolonged darkness, coupled by the deep-water currents radiating from Antarctica, may support an Antarctic origin for deep-sea taxa. Although evidence for Antarctic deep-sea origins has been found for octopods (Strugnell et al. 2008) and isopods (Hessler and Thistle 1975), no studies have yet addressed a potential Antarctic origin for hadal liparids.

Future phylogenetic work will also require careful consideration of taxonomic classifications, which are in need of revision across the snailfishes. With the collection of additional hadal liparid species, the two liparid genera *Notoliparis* and *Pseudoliparis—*which overlap in most taxonomic and ecological characteristics—should likely be synonymized (Gerringer et al. 2017b). Similar research has begun for hadal amphipods, which shows complex connectivity patterns and species overlap, particularly in the genus *Hirondellea* (Ritchie et al. 2015). It will be worthwhile to continue to explore how connected or distinct individual hadal trenches are from one another and the waters above, and whether these patterns vary across taxa. While it may be tempting to think of hadal trenches as remote, isolated habitats, evidence increasingly shows that trenches are closely tied to the surrounding ocean systems, even accumulating man-made pollutants at high concentrations (Jamieson et al. 2017).

Interestingly, there are now a few hadal trenches known to have more than one species of apparently endemic hadal snailfish. The Japan Trench has both *Pseudoliparis belyaevi* and *P.amblystomopsis* (Andriashev and Pitruk 1993). Recent work has revealed two genetically distinct populations in the Kermadec Trench—*N.kermadecensis* and *Notoliparis stewarti* (Stein 2016; Gerringer et al. 2017b). There are also two snailfish populations in the Mariana Trench—*P.swirei* and the ethereal snailfish, which remains uncollected (Linley et al. 2016; Gerringer et al. 2017b). It is possible that the potential pelagic larval stage proposed from otolith microchemistry (Gerringer et al. 2018) provides a dispersal mechanism for these populations, for example for *P. amblystomopsis*, which is found in both the Japan and Kuril–Kamchatka trenches (Andriashev and Pitruk 1993). In the case of the ethereal snailfish and *P. swirei*, it is possible to envision distinct ecological niches. In form, these two fishes are very distinct, with the delicate ethereal snailfish almost certainly representing an undescribed genus. *Pseudoliparis swirei* is an active predator, feeding on amphipods and decapods in the water (Gerringer et al. 2017d). No ethereal snailfish specimens have been collected, so stomach content and stable isotope analyses have not been possible, but one fish was observed moving headfirst into the sediment in what appears to be a feeding behavior (Linley et al. 2016), suggesting that it may rely instead on benthic invertebrates. However, for *P. belyaevi* and *P. amblystomopsis*, and for *N. kermadecensis* and *N. stewarti*, there is no obvious driver of speciation. Indeed, they are indistinguishable in video. Collections of *N. kermadecensis* and *N. stewarti* have revealed no obvious distinctions in trophic ecology, pressure adaptation, or life history (Gerringer et al. 2017c, 2017d, 2018), despite the presence of two clades based on mitochondrial gene sequences (Gerringer et al. 2017b). Perhaps these populations were once geographically isolated and the genetic differences reflect some period of extended isolation in the past.

Evolution into the deep sea naturally requires adaptation to high hydrostatic pressure. However, reconstructing these evolutionary processes requires consideration of an important factor: pressure and temperature have interacting effects on biomolecules (e.g., Dahlhoff and Somero 1991; Somero 1992). An interesting result of pressure–temperature interaction is that a change in the temperature of the deep ocean would have profound and complex effects on organismal responses to pressure. As one example for consideration, the proposed depth limit of ∼8200 m (Yancey et al. 2014) would likely be temperature dependent, as both pressure and temperature affect volume changes associated with physiological processes (reviewed by Somero 1992). Indeed, colonization of the deep sea is believed to have occurred during warmer temperatures (Priede and Froese 2013), which may have allowed easier adaptation to higher pressures. Eventually, it may be possible to develop a model that quantifies pressure and temperature interactions on a molecular level, allowing something like the agricultural growing degree-day, which relates growth rates to temperature (e.g., Neuheimer and Taggart 2007)—a growing-degree-day-MPa—to be applied in discussions of the rates of life and adaptation to different pressure–temperature regimes (Gerringer et al. 2017c).

## Future directions

Although recent decades have marked significant advancements in understanding of the hadal zone, there is still much that remains unknown about these habitats. Snailfishes have been found in at least six hadal trenches globally (Fig. 1 and Table 2). It is likely that additional hadal snailfish populations exist in trenches that have not yet been thoroughly explored. However, there is at least one trench, the New Hebrides, that does not appear to house an endemic hadal snailfish population (Linley et al. 2017). The reasons for this absence are not fully understood, but may relate to a distinct water mass input compared with trenches like the Kermadec, Mariana, and Japan, or to differences in surface productivity of overlying waters (Linley et al. 2017). Future exploration of trenches near the New Hebrides, such as the Vitjaz, Bougainville, San Cristobal, and New Britain, would help to clarify these drivers.

Hadal science in the last 50 years has been largely exploratory. The next phase of hadal research should strive for greater temporal and spatial understanding of deep-sea trenches, including of the fish community. From the videos, images, and samples of the hadal zone collected so far, it is already clear that there is great topographic and faunal heterogeneity within individual trenches and between trenches (Stewart and Jamieson 2018). The factors driving these changes warrant further exploration. Further, much of the work done at hadal depths has been on bait-attending fauna, due to technical constraints. Although useful, free-falling lander equipment provides a certain biased view of the community (e.g., Jamieson 2018). Alternative sampling and exploration methods, including remotely operated vehicles, will need to be employed to gain a more holistic view of hadal fauna and their physiology and ecology. In the future, *in situ* experiments would provide valuable additional information about life in the hadal zone. For example, existing methods of measuring metabolic rates in deep-living fishes (e.g., Drazen et al. 2015) have been complicated by pressure effects on enzyme activities (Gerringer et al. 2017c). Studies using *in situ* oxygen consumption measurements on hadal fishes could be compared with shallower-living taxa to disentangle the roles of pressure, temperature, and food supply on metabolic rates that existing methods.

Additional exploration of abyssal ecosystems will also be needed to understand what makes the hadal zone distinct. Much of the work comparing the hadal liparids to abyssal fishes has been conducted using the prominent abyssal macrourids *C.armatus* and *C. yaquinae* as representative abyssal taxa. The macrourids are certainly an important abyssal group, with representatives in most ocean basins at a broad range of depths (Wilson and Waples 1983; Jamieson et al. 2009). Because there is a diversity of fishes living at abyssal depths, however, this is an oversimplification of the interactions at play in structuring the fish communities at abyssal and hadal depths. In particular, this view likely underrepresents the role of the cusk eels (Ophidiidae). Ophidiids are a largely understudied group, though they are wide-ranging and likely important members of the abyssal fish community (Nielsen and Merrett 2000; Uiblein et al. 2008; Nielsen and Møller 2011; Linley et al. 2017). To more thoroughly compare abyssal and hadal fishes, future research should focus on the biogeography, ecology, and physiology of the deep-dwelling cusk eels—to address their role in the abyssal ecosystem. Other abyssal fish families could also be compared in more detail in future work, including the eelpouts (Zoarcidae), cutthroat eels (Synaphobranchidae), and tripodfishes (Ipnopidae), the last of which are very poorly studied as they are not attracted to baited cameras or traps. Furthering our understanding of deep bathyal and abyssal liparids would also provide insight into the evolution of fishes into the deep sea.

Hadal science is by necessity highly collaborative and interdisciplinary work. It will take continued cooperation between a host of scientists and engineers across borders to further our understanding of these communities in the ocean’s greatest depths and their role in the global ocean ecosystem.

## Supplementary Material

Supplementary_Material_obz004
