# Supplementary material for: On the Success of the Hadal Snailfishes
Source: Integr Org Biol. 2019 Mar 23;1(1):obz004. doi: 10.1093/iob/obz004 (PMC7671157; doi:10.1093/iob/obz004)
Supplement: Supplementary_Material_obz004 [file supplementary_material_obz004.docx]

**Supplementary Note 1**

On the Success of the Hadal Snailfishes

M.E. Gerringer

*Integrative Organismal Biology*

doi: 10.1093/iob/obz004

With limited deep-sea sampling opportunities relative to the vastness of the deep-sea environment, knowing the true bathymetric distributions of deep-sea fishes is a difficult task. This problem is also complicated by a number of specious records that stem from misidentifications and pelagic contaminants caught in open nets. For example, the record of the ophidiid *Abyssobrotula galatheae* reported from over 8,000 m comes from an open trawl, and is a far reach from the common records of the species at depths ~3,000 m (Fujii et al., 2010; Jamieson and Yancey, 2012; Nielsen, 1977). **Figures 4** excludes a record of the ophidiid *Echiodon neotes*, reported from 8,300 m, that is understood to most likely be a pelagic capture from shallower depths by experts on the group (Nielsen et al., 1999). The deep-sea smelts, in the family Bathylagidae, have also been excluded. A few reports list collections from great depths, including *Bathylagus pacificus* at 7,700 m and *Pseudobathylagus milleri* at 6,600 m (Froese and Pauly, 2014), but these are most likely pelagic contaminants from near ~1000 m, where the overwhelming abundance of collections of these groups occur (Shinohara et al., 1994). Additional details and examples of improbable collection records are discussed in depth elsewhere (Fujii et al. 2010; Jamieson and Yancey 2012; Linley et al. 2016b).
